# Tivantinib (ARQ 197) affects the apoptotic and proliferative machinery downstream of c-MET: role of Mcl-1, Bcl-xl and Cyclin B1

**DOI:** 10.18632/oncotarget.4240

**Published:** 2015-06-10

**Authors:** Shuai Lu, Helga-Paula Török, Eike Gallmeier, Frank T. Kolligs, Antonia Rizzani, Sabrina Arena, Burkhard Göke, Alexander L. Gerbes, Enrico N. De Toni

**Affiliations:** ^1^ Medizinische Klinik und Poliklinik 2, Klinikum der Universität München, Campus Grosshadern, Munich, Germany; ^2^ Department of Gastroenterology, Endocrinology and Metabolism, University Hospital of Marburg, Philipps-University of Marburg, Marburg, Germany; ^3^ Department of Internal Medicine and Gastroenterology, HELIOS Klinikum Berlin-Buch, Berlin, Germany; ^4^ Department of Oncology, University of Torino, Candiolo, Torino, Italy; ^5^ Candiolo Cancer Institute-FPO, IRCCS, Candiolo, Italy

**Keywords:** HCC, targeted therapies, c-MET, apoptosis

## Abstract

Tivantinib, a c-MET inhibitor, is investigated as a second-line treatment of HCC. It was shown that c-MET overexpression predicts its efficacy. Therefore, a phase-3 trial of tivantinib has been initiated to recruit “c-MET-high”patients only. However, recent evidence indicates that the anticancer activity of tivantinib is not due to c-MET inhibition, suggesting that c-MET is a predictor of response to this compound rather than its actual target. By assessing the mechanisms underlying the anticancer properties of tivantinib we showed that this agent causes apoptosis and cell cycle arrest by inhibiting the anti-apoptotic molecules Mcl-1 and Bcl-xl, and by increasing Cyclin B1 expression regardless of c-MET status. However, we found that tivantinib might antagonize the antiapoptotic effects of c-MET activation since HGF enhanced the expression of Mcl-1 and Bcl-xl. In summary, we show that the activity of tivantinib is independent of c-MET and describe Mcl-1, Bcl-xl and Cyclin B1 as effectors of its antineoplastic effects in HCC cells. We suggest that the predictive effect of c-MET expression in part reflects the c-MET-driven overexpression of Mcl-1 and Bcl-xl in c-MET-high patients and that these molecules are considered as possible response predictors.

## INTRODUCTION

Tivantinib (ARQ 197) is being investigated in clinical trials as selective orally available c-MET inhibitor [1]. Administered as a single agent in a second-line treatment setting, tivantinib was shown to prolong significantly the time to progression and the survival of hepatocellular carcinoma (HCC) patients in a randomized, placebo-controlled, phase 2 clinical trial [2, 3]. Analysis of c-MET expression showed a clear advantage in overall survival in patients bearing tumors exhibiting a highly positive staining for c-MET but not in “MET-low” patients. The administration of tivantinib is thus regarded as a promising *bona fide* biomarker-based therapy of HCC. Consequently, patients' selection in the ongoing phase 3 METIV-HCC trial of tivantinib is based on the detection of high expression of c-MET in tumor biopsies. The predictive value of c-MET in determining survival improvement in patients on tivaninib was recently confirmed by a subgroup analysis of a randomized controlled trial in patients with non-squamous, non-small cell lung cancer [4, 5]. Although the clinical efficacy of tivantinib in c-MET-high patients in the two aforementioned trials suggests that its anticancer activity is determined by its capability to inhibit c-MET, several *in vitro* studies published very recently challenged this notion by showing that this compound exerts a remarkable cytotoxic effect in several cell lines without affecting the kinase activity of this receptor. These studies questioned the rationale for the use of this compound in c-MET-high patients [6–9] and raised the issue of whether c-MET represents a response predictor of tivantinib rather than its actual target [10, 11]. In spite of the clinical relevance of this issue, the mechanisms of action of tivantinib as well as those determining the predictive value of c-MET expression still remain to be elucidated.

In the attempt to provide an answer to this question, we decided to investigate the so far still unclear intracellular mechanisms of action of tivantinib on cell death and cell cycle progression, and to assess how their regulation is influenced by this compound in cell lines exhibiting different c-MET expression status [12, 13].

## RESULTS

### Tivantinib causes a strong loss of cell viability and of colony forming capability in a wide panel of cell lines from gastrointestinal tumors

**Figure 1:**
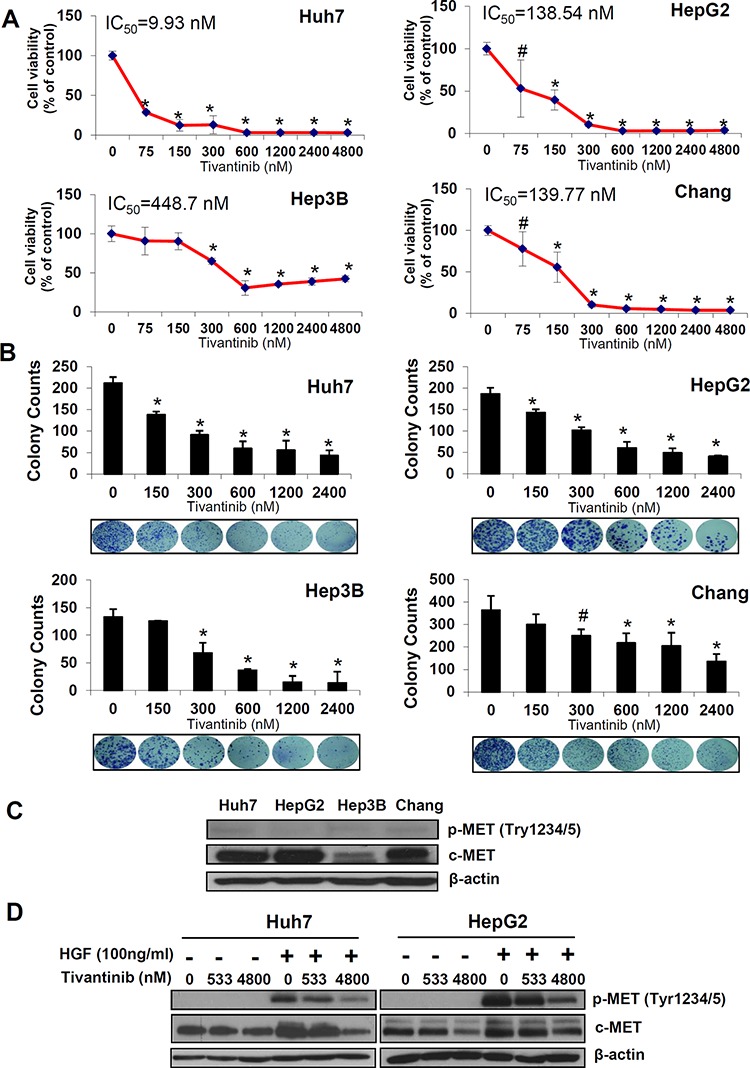
Tivantinib reduces cell viability and colony formation of HCC cells **A.** Effect of increasing concentrations of tivantinib on cell viability as judged by Sybr green assays in the indicated cell lines. **B.** Colony forming assay showing number of colonies and representative figures of the indicated cell lines. Results are expressed as mean and standard deviation of 3 independent experiments each conducted in triplicate. *p < 0.01; #*p* < 0.05 vs. control treated cells. **C.** Western blot analysis of c-MET and p-c-MET in the indicated cell lines. **D.** Effect of tivantinib on phosphorylation status of c-MET in Huh7 and HepG2 cells after administration of the c-MET ligand HGF. For this experiment, cells were incubated with tivantinib for 24 hours; HGF was added at the concentration of 100 ng/ml for 10 minutes before the cells were harvested.

The effect of tivantinib on cell viability was assessed in a wide panel of cell lines exhibiting different levels of c-MET expression including 4 HCC cell lines (Fig. 1A), one cholangiocellular carcinoma cell line, and three additional cancer cell lines from tumors of gastrointestinal origin (Fig. S1). Tivantinib caused a dose dependent loss of cell viability with IC_50_ values comprised between 9.9 nM (Huh7) and 448 nM (Hep3B). These results were clearly confirmed by colony forming assays showing a reduction in the number and size of colonies in cells treated with tivantinib (Fig. 1B, Fig. S1B). As shown in Figure 1C-1D, the effect of tivantinib on phosphorylated c-MET was not evident in unstimulated cells due to low basal level of p-c-MET; however, administration of tivantinib with the c-MET ligand HGF caused a decrement of total c-MET as well as of its phosphorylated form in Huh7 or HepG2 cells (Fig. 1D). This phenomenon, which was also reported previously [6], shows that the effect of tivantinib on overall c-MET largely accounts for the observed decrease of c-MET phosphorylation.

### Tivantinib enhances apoptosis by inhibiting the mitochondrial regulators of apoptosis Mcl-1 and Bcl-xl

**Figure 2:**
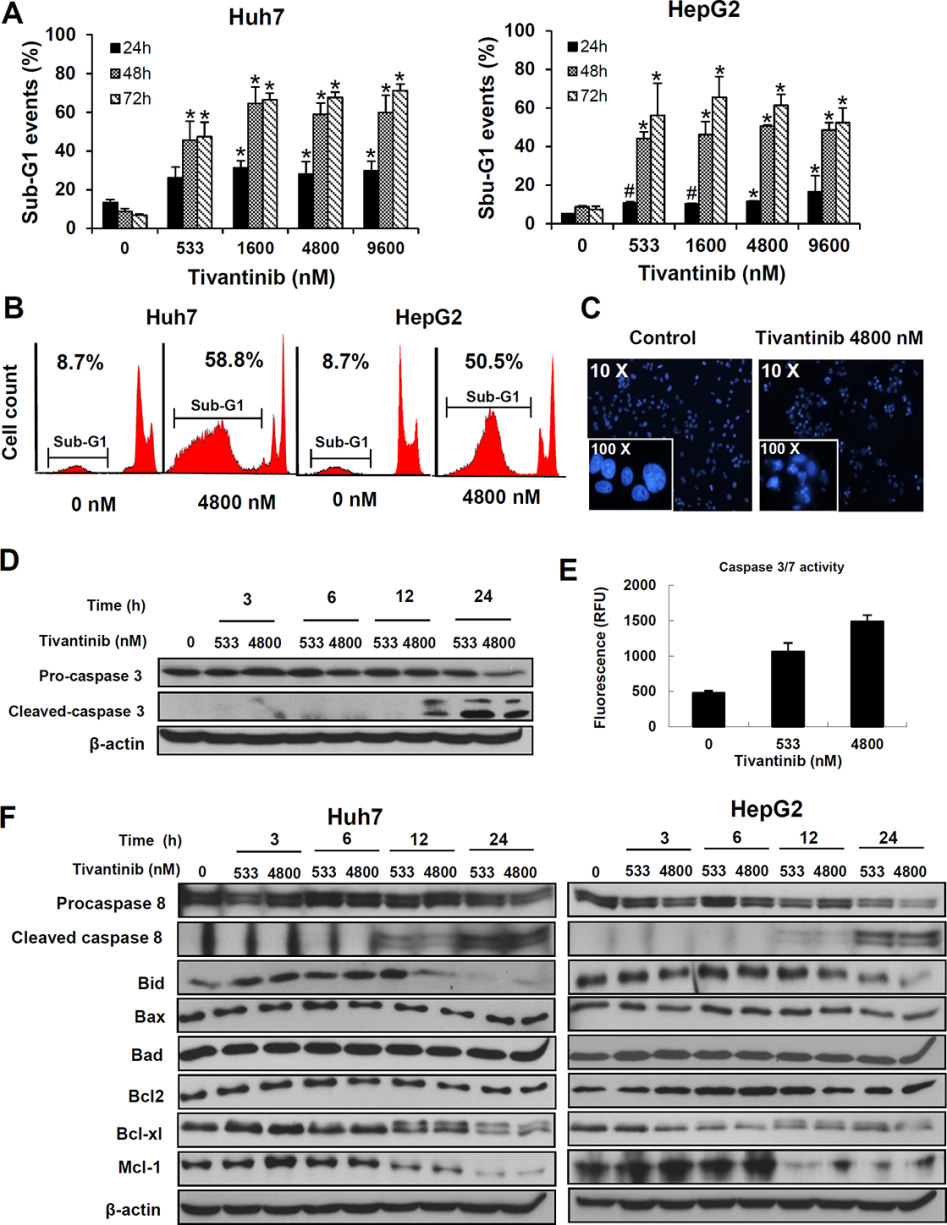
Tivantinib causes apoptosis by activating the mitochondrial apoptotic pathway **A** and **B.** Quantification (A) and typical FACS patterns (B) of apoptotic cells based on the count of the cell fraction showing subdyploid DNA content (sub-G1 fraction) after propidium iodide staining. *p < 0.01; #*p* < 0.05 in comparison to control treated cells. **C.** Fluorescence microscopy features after Hoechst staining showing typical chromatin condensation and nuclear fragmentation of Huh7 cells. **D** and **E.** Assessment of caspase 3 cleavage by western blot (D) and of caspase 3/7 activation as determined by fluorimetric assessment in HepG2 cells after 24 hours (E). **F.** Time kinetic of the expression of different regulators of apoptosis was performed by incubating Huh7 or HepG2 cell lines for the indicated time.

To assess the mechanisms underlying the decrease in cell viability caused by tivantinib, we subsequently investigated its effect on apoptosis. As shown by the increasing sub-G1 cell fraction at FACS analysis after PI staining (Fig. 2A, S2A) tivantinib caused a dose- and time-dependent increase of apoptosis. Induction of apoptosis was observable at the concentration of 533 nM and most cells showed features of apoptosis at a concentration of 1.6 μM after 48 hours of incubation (Fig. 2A) with chromatin condensation and nuclear fragmentation at Hoechst staining (Fig. 2C). Accordingly, progressive time- and dose-dependent increase of caspase 3 cleavage (Fig. 2D, S2B, S3A), increased caspase 3/7 activation (Fig. 2E, S3B) and cleavage of PARP (Fig. S3) were observed.

To further investigate the mechanisms underlying tivantinib-induced apoptosis, the effects of tivantinib on the two major pro-apoptotic signaling pathways-the extrinsic and the intrinsic apoptotic pathways-were assessed. Analysis of Caspase 8 showed a time and dose-dependent cleavage of Pro-caspase 8 and Bid (Fig. 2F), indicating an increased activation of the receptor-mediated apoptotic pathway. Accordingly, administration of tivantinib sensitized Huh7 and HepG2 cells to the action of the TRAIL-R2 agonistic antibody tigatuzumab, which initiates the extrinsic apoptotic pathway dependently on Caspase 8 recruitment [14] (Fig. S4).

Analysis of the activation of the intrinsic apoptotic pathway showed that tivantinib additionally causes the downregulation of the antiapoptotic molecules Bcl-xl and Mcl-1 (Fig. 2F).

**Figure 3:**
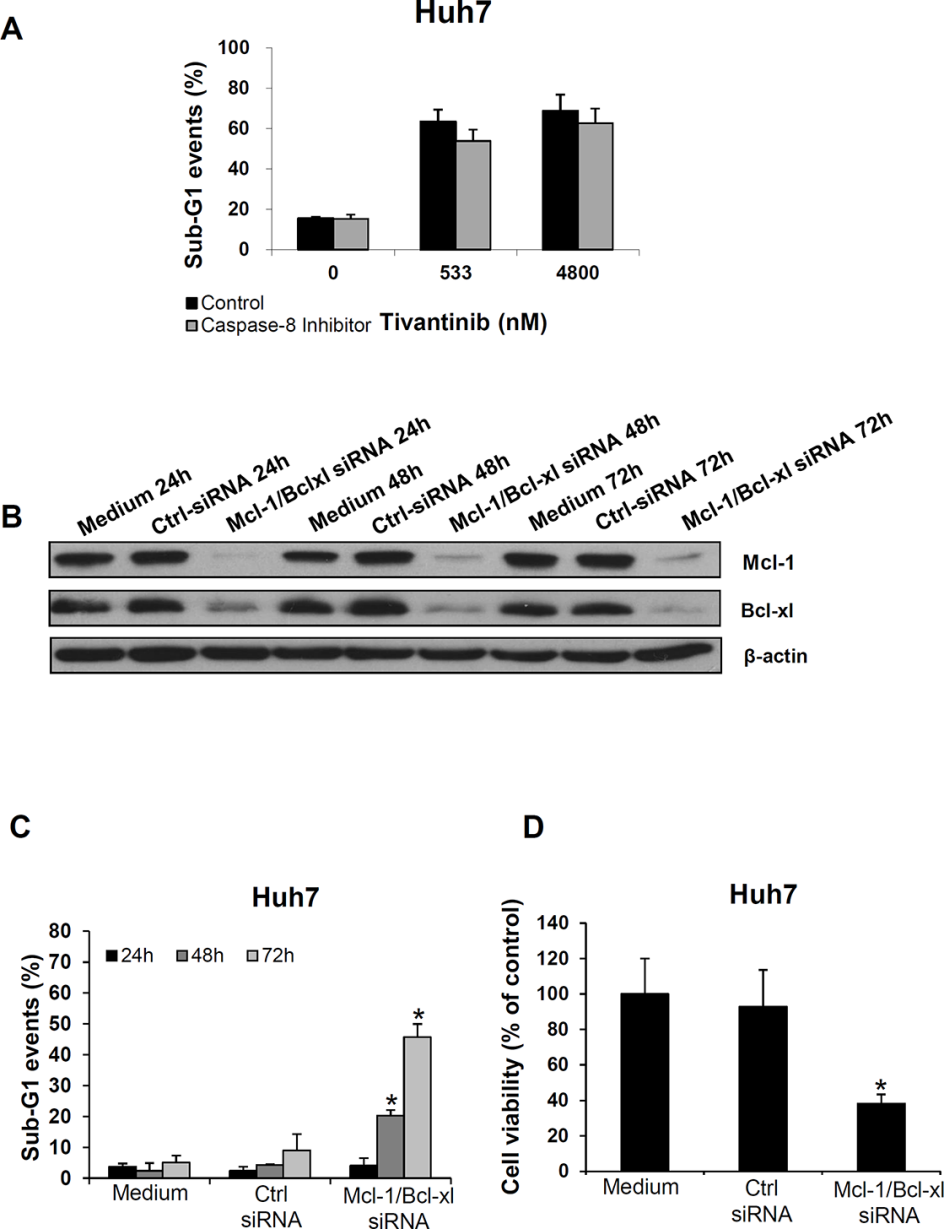
Mcl-1 and Bcl-xl play a functional role in determining the apoptotic effect of tivantinib **A.** Effect of Caspase 8 inhibition on tivantinib-mediated apoptosis. FACS analysis was performed to assess nuclear fragmentation 72 hours after administration of tivantinib in the presence or absence of the Caspase8 inhibitor Z-IETD-FMK. **B.** Effect of Mcl-1 and Bcl-xl silencing by co-transfection of specific siRNA oligonucleotide sequences in Huh7 cells on expression of Bcl-xl and Mcl-1 (each at the concentration of 50 nM); Medium or non-coding siRNA (Ctrl-siRNA) were used as control. **C** and **D.** FACS analysis of apoptosis (C), and cell viability (D) after transfection of siRNA targeting Mcl-1 and Bcl-xl. *p < 0.01, in comparison to cells transfected with non-coding siRNA.

To assess the respective functional relevance of the extrinsic and intrinsic pathway activation in determining the effect of tivantinib, Huh7 cells were co-incubated with tivantinib and the Caspase 8 inhibitor Z-IETD-FMK; as shown in Fig. 3A, inhibition of Caspase 8 only marginally reduced the proapoptotic effect of tivantinib. In contrast, silencing of Mcl-1 and Bcl-xl by simultaneous co-transfection of specific siRNA sequences in Huh7 cells led to a progressive increase of apoptosis and consequent loss of cell viability over 72 hours (Fig. 3B–3D). Analysis of cell cycle distribution in these samples showed no cell cycle arrest in consequence of Mcl-1 and Bcl-xl silencing (data not shown).

Due to the negligible effect of Caspase 8 inhibition on apoptosis, these data indicate that tivatinib induces apoptosis by shifting the balance between the pro- and anti-apoptotic mitochondrial regulators of the intrinsic signaling pathway (Fig. 2F).

### Tivantinib-mediated accumulation of Cyclin B1 is associated with a G2/M cell cycle arrest

**Figure 4:**
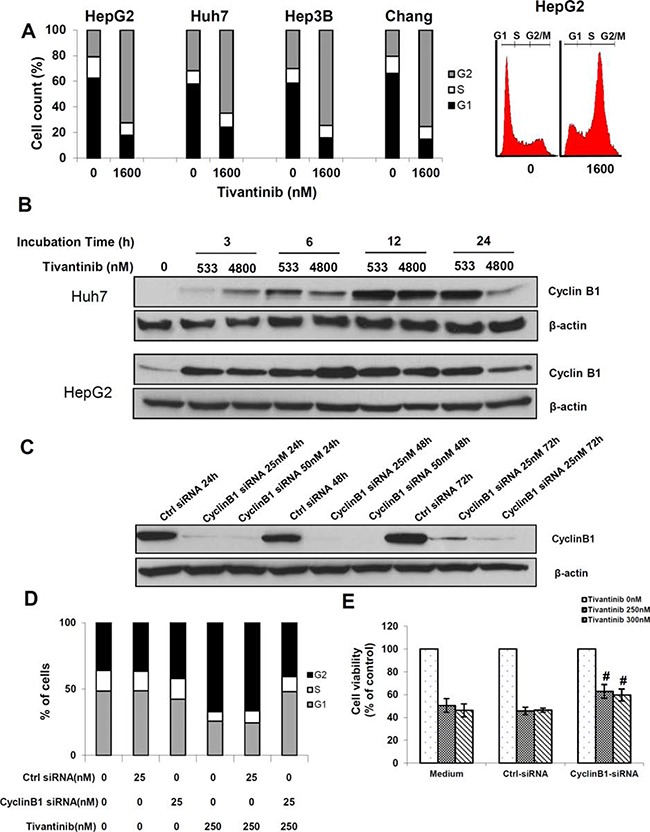
Tivantinib causes a Cyclin B1-dependent G2 cell cycle arrest **A.** Cell cycle quantification and typical flow cytometry pattern of G2/M cell cycle arrest of the indicated HCC cell lines after incubation with tivantinib for 24 hours and PI staining. **B.** Time kinetic of Cyclin B1 activation. **C.** Effect of Cyclin B1 silencing by specific siRNA (Cyclin B1-siRNA) on Huh7 cells. Non-coding siRNA (Ctrl-siRNA) was used as control. **D** and **E.** Effect of Cyclin B1 silencing on cell cycle (D) and cell viability (E) of Huh7 cells. After overnight incubation, cells were transfected with Cyclin B1-targeting siRNA for 24 h, and then treated with tivantinib for 1 hour before harvesting. Cellular viability was assessed by MTS assay in Huh7 cells after 48 hours. #*p* < 0.05 vs. cell incubated in medium only or non-coding siRNA.

Since tivantinib was reported to induce a G2-cell cycle arrest, we subsequently performed an analysis of the cell fraction in different phases of the cell cycle by FACS (Fig. 4A). Administration of tivantinib caused a G2/M cell cycle arrest in all cell lines assessed, with a corresponding decrease of the fraction of cells in G1 phase. For example, the fraction of cells in G1, S and G2/M phase in vehicle treated Huh7 cells were 57.9 ± 4.1%, 10.4 ± 5.9%, and 31.7 ± 9.8% respectively. After 24 h incubation with tivantinib at the concentration of 1.6 μM the fractions of cells in the different phases of cell cycle were 24.1% ± 2.8, 11.1 ± 2.0%, 64.9 ± 9.8% respectively (*P* < 0.01 - Fig. 4A). Similar effects were observed in several cell lines from colon cancer, cholangiocellular and pancreatic cancer cells (data not shown). To determine the mechanisms underlying the G2/M cell cycle arrest caused by tivantinib, expression of Cyclin B1, which is known to regulate cellular transition at the G2/M checkpoint [15], was assessed. Western blot analysis showed a strong increase of Cyclin B1 as soon as 3 hours after incubation (Fig. 4B). To assess whether increase of Cyclin B1 is functionally related to the cycle arrest caused by tivantinib, cells were transfected by specific Cyclin B1-targeting siRNA. As shown in Fig. 4C, silencing of Cyclin B1 prevented the G2/M cell cycle arrest (Fig. 4D) and significantly reduced the loss of cell viability caused by tivantinib (Fig. 4E). These data indicate that a Cyclin-B1 dependent G2/M cell cycle arrest concurs with the induction of apoptosis to determine the antineoplastic effect of tivantinib.

### The antiproliferative effect of tivantinib is independent of c-MET but affects targets downstream of c-MET

**Figure 5:**
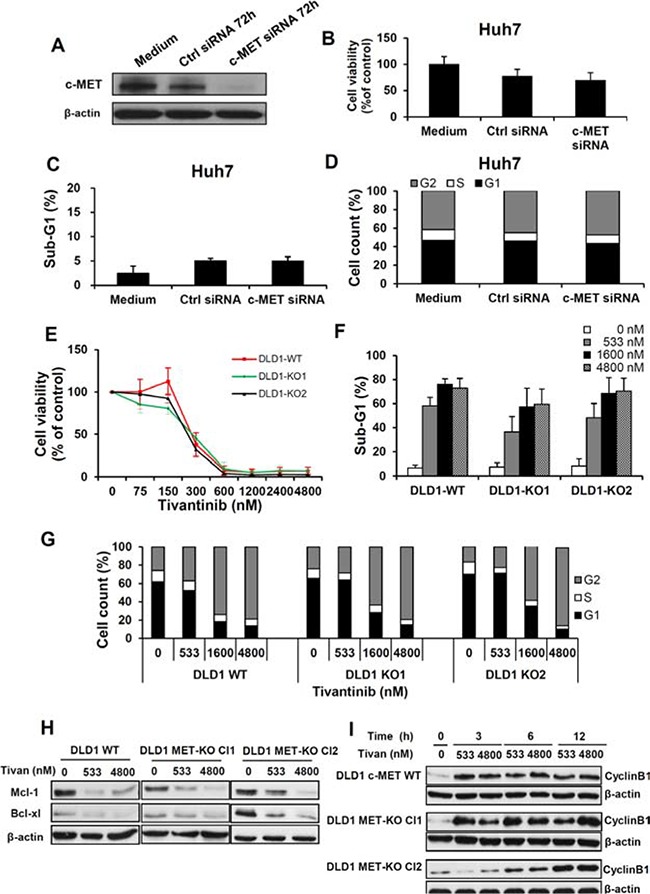
The antineoplastic effect of tivantinib is not dependent on c-MET **A, B, C** and **D.** Effect of c-MET silencing on the expression of c-MET (A) on cell viability (B), apoptosis (C) and cell cycle (D), in Huh7 cells 72 hours after transfection of specific (c-MET-siRNA) or non-coding siRNA (ctrl-siRNA). **E, F, G, H** and **I.** Effect of tivantinib on *c-MET* wild-type DLD1 colorectal cancer cell lines or in *c-MET* exon 16 knock-out DLD1 (two different clones) as determined by viability assay 6 days after incubation with tivantinib (E), assessment of apoptosis (F) and cell cycle analysis (G) 48 hours after incubation with tivantinib. Western Blot analysis (H) of Mcl-1 and Bcl-xl after 24 hours incubation with tivantinib andtime kinetic of Cyclin B1 activation (I) in these cell lines.

After describing Bcl-xl, Mcl-1 and Cyclin B1 as functional targets of tivantinib in determining apoptosis and cell cycle arrest, we assessed whether the influence of tivantinib on these molecules is dependent on its efficacy as inhibitor of c-MET, as the predictive significance of this receptor in clinical trials seems to suggest [2, 16]. To this aim, we assessed the effects of c-MET silencing by siRNA transfection in Huh7 cells; additionally, we compared the effect of tivantinib on DLD1 cells, which express the native form of c-MET vs. that on syngenic DLD1 c-MET exon 16 KO cell lines, which express a genetically modified variant of c-MET lacking the binding site of tivantinib. Silencing of c-MET by siRNA failed to increase apoptosis and cell cycle arrest in Huh7 cells and only modestly affected cell viability in these cells (Fig. 5A–5D). We also observed that the effect of tivantinb on cell viability, cell cycle and apoptosis in DLD1 wild type (WT) cells was indistinguishable from that observed in two independent c-MET exon 16 knock out cell clones (KO1 and KO2) (Fig. 5E–5G). Western blot analysis of these DLD1 cell clones showed that the expression of Mcl-1 and Bcl-xl was reduced and that of Cyclin B1 increased in both wild type and c-MET-exon 16 knock out DLD1 cells (Fig. 5H and 5I) upon incubation with tivantinib. The biological effects of tivantinb and the associated molecular changes observed in Huh7 and HepG2 cell lines were thus reproducible in 2 additional cell clones derived from DLD1 colorectal cancer cells and were independent of the expression of c-MET; this suggests that the antineoplastic activity of tivantinib is largely independent of its effect on this receptor.

We subsequently hypothesized that the significance of c-MET as predictor of the outcome of patients on tivantinib may be related to the fact that Mcl-1, Bcl-xl or Cyclin B1 are downstream targets of c-MET, and that the efficacy of tivantinib in tumors overexpressing c-MET may reflect the effect of this compound on c-MET-driven overexpression of these molecules.

**Figure 6:**
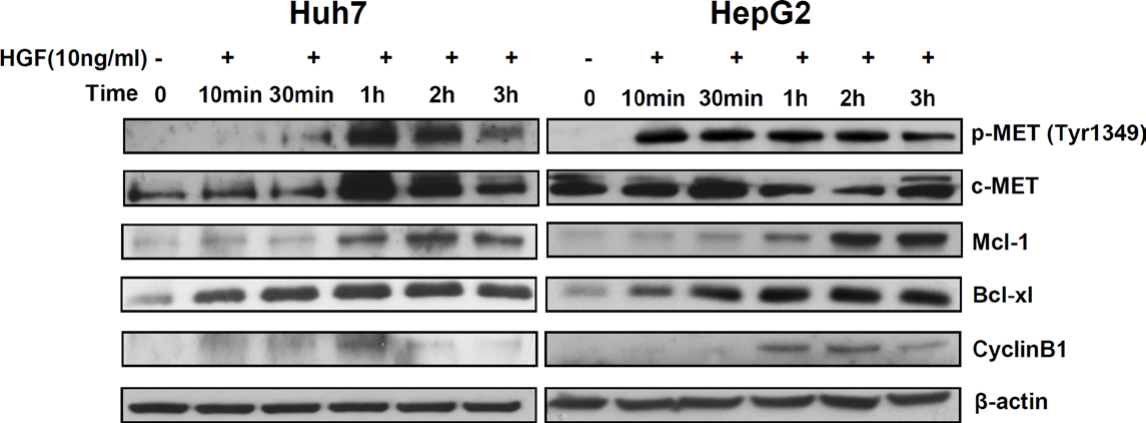
Mcl-1 and Bcl-xl are regulated by stimulation of c-MET Time kinetic of phospho-MET, Mcl-1, Bcl-xl and Cyclin B1 at western blot analysis. Huh7 and HepG2 cells were incubated with 10 ng/ml human recombinant HGF and collected at the indicated time points.

To assess this possibility, a time kinetic of c-MET, Bcl-xl, Mcl-1 and Cyclin B1 expression was performed in Huh7 and HepG2 cells after incubation with HGF, which is the only known ligand of c-MET. This analysis showed that activation of c-MET leads to increased phosphorylation of c-MET (Tyr1349) and to increased Mcl-1 and Bcl-xl. However, a slight increase of Cyclin B1 could also be observed upon c-MET stimulation (Fig. 6). These data show that while tivantinib might antagonize the effect of c-MET-mediated expression of Mcl-1and Bcl-xl, the effect of tivantinib Cyclin B1 might not be predictable by the expression of c-MET.

## DISCUSSION

c-MET inhibitors are considered a promising therapeutic option for tumours exhibiting c-MET-status alterations [17]. Tivantinib prolonged time-to-progression and overall survival among c-MET diagnostic-high HCC patients as a second line treatment after failure of a first line sorafenib treatment [2, 3] Therefore, only patients selected according to a “c-MET-high” status are recruited in the ongoing phase-3 study of tivantinib in HCC [3]. Unexpectedly, the concept of tivantinib as c-MET inhibitor has been recently challenged, and it has been suggested that c-MET might represent a predictor of efficacy rather than the actual target of this compound [6–9]. In this paper we addressed this issue 1) by investigating the so far unknown molecular mechanisms underlying apoptosis and cell cycle induced by tivantinib and 2) by assessing whether these effects are specifically dependent on the effect of tivantinib on c-MET. These topics and their clinical implications are discussed below.

### Molecular mechanisms underlying the effect of tivantinib on apoptosis and cell cycle

Since tivantinib was previously shown to induce apoptosis and G2/M-cell cycle arrest, we first assessed its effect on crucial mechanisms of apoptosis and cell cycle control. Assessment of apoptosis showed that tivantinb causes cleavage of Procaspase-8 followed by the cleavage of Bid. Cleavage of Caspase 8 is a hallmark of the activation of the extrinsic apoptotic pathway, which is initiated at the cell membrane in consequence of the stimulation of the “death-receptors” such as TRAIL-R1 and –R2 or CD95. Accordingly, administration of tivantinib sensitized several cancer cell lines to the action of the TRAIL-R2 agonistic antibody tigatuzumab (Fig. S4). Nevertheless, inhibition of Caspase 8 only marginally affected apoptosis induced by tivantinib indicating that activation of the extrinsic signalling pathway plays a marginal role in triggering apoptosis observed in our system.

As we assessed the effect of tivantinib on the intrinsic signaling pathway we observed a strong decrease of the antiapoptotic proteins Bcl-xl and Mcl-1. The intrinsic pathway is regulated by the balance between pro-apoptotic molecules (such as Bax, Bak, Bim and Bid) and antiapoptotic molecules (such as Bcl-2, Bcl-xl and Mcl-1). Mcl-1 and Bcl-xl are Bcl-2-related proteins which bind to Bax and Bak, thereby blocking their pro-apoptotic interaction with the outer surface of the mitochondria [18]. The functional relevance of Bcl-xl and Mcl-1 in determining apoptosis mediated by tivantinib was confirmed by si-RNA against Bcl-xl and Mcl-1 which caused apoptosis to an extent comparable with that observed upon tivantinib administration. The effect on Mcl-1 of tivantinib on Mcl-1 is in keep with previous findings showing that CKD inhibitors trigger apoptotic cell death by inhibiting Mcl-1 [19]. These data show that while cleavage of Caspase 8 and activation of the extrinsic apoptotic pathway might play a role in determining the effect of tivantinib *in vivo* e.g. by affecting the response to endogenous TRAIL, tivantinib causes apoptosis principally by shifting the balance between the mediators of the mitochondrial pathway by inhibiting Bcl-xl and Mcl-1.

In addition, we observed a strong increase of Cyclin B1 as soon as 3 hours after administration of tivantinib. Cyclin B1 controls the G2-M cell cycle transition [20, 21] and its elevation was shown to cause mitotic delay in Hela cells [15, 22]. In agreement with these previous reports, we found that silencing of Cyclin B1 reversed the G2/M cell cycle arrest caused by tivantinib and significantly reduced its effect on cell viability.

### c-MET expression and response to tivantinib

Experiments conducted with *c-MET* exon 16 KO DLD1 cell lines, which lack the tivantinib-binding site of c-MET, showed that the effect of tivantinib in these cell lines was indistinguishable from that observed in parental-Wild Type DLD1 cell lines. Accordingly, tivantinib led to the downregulation of Bcl-xl, Mcl-1 and to the upregulation of Cyclin B1 independently of the c-MET status of these cell clones. Moreover, silencing of c-MET by specific siRNA failed to reproduce the effect of tivantinib on apoptosis and cell cycle. Administration of tivantinib caused a decrement of total c-MET and, to the same extent, of its phosphorylated form, indicating that the decrease of overall c-MET largely accounts for the observed decrease of c-MET phosphorylation. Nevertheless, our results show that the antineoplastic activity of tivantinib is largely independent of its effect as c-MET inhibitor.

This discrepancy between our data and the predictive effect of c-MET expression in the clinical setting might be due to differences related to the higher complexity of the mechanisms of action of tivantinib *in vivo*, e.g. those underlying invasion and metastasis formation [23], and to microenvironmental changes related to previous administration of sorafenib in these patients. Such hypothesis would be supported by the reported role of c-MET in hypoxic conditions and in resistance to anti-angiogenic therapy [24, 25]. Nevertheless, the remarkable effect of tivantinib on cell viability, cell cycle and apoptosis observed in our experiments suggests that these effects account for the antineoplastic effects of tivantinib observed in the clinical setting.

To find possible explanations for the predictive relevance of c-MET in clinical trials, we hypothesized that Bcl-xl, Mcl-1 and Cyclin B1 are downstream targets of c-MET, and that the efficacy of tivantinib on tumors overexpressing c-MET may reflect the effect of this compound on c-MET-driven overexpression of these molecules. Stimulation of c-MET by HGF showed a clear increase of Bcl-xl and Mcl-1 suggesting that tivantinib might antagonize the effect of c-MET-dependent expression of these molecules in cells overexpressing this receptor regardless of the inhibitory effect of tivantinib on the kinase activity of c-MET.

**Figure 7:**
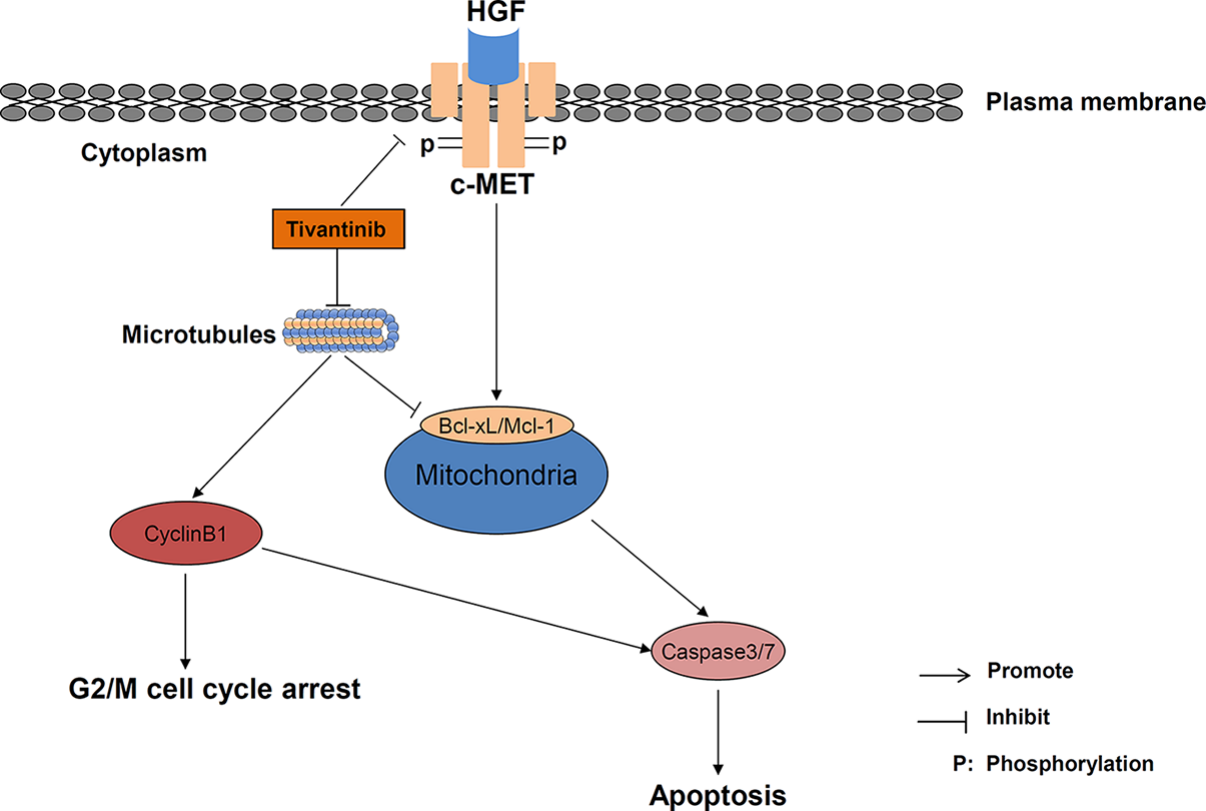
Schematic diagram of the postulated interaction between tivantinib and intracellular mechanisms of apoptosis and cell cycle regulation

Although the mechanisms by which tivantinib causes such molecular alterations still remains elusive, a possible answer to this issue might be provided by the recent works published by Basilico [6], Katayama [7], and their colleagues. These authors found that tivantinib exerted a cytotoxic effect in several cell lines without affecting the function of c-MET by causing microtubule stabilization or impairment of tubulin polymerization, respectively. Interestingly, microtubule-damaging agents are known to increase Cyclin B1 and to activate the spindle assembly checkpoint, and degradation of Mcl-1 has been shown to occur as consequence of administration of microtubule-targeting agents [26]. It is thus likely that tivantinib affects cell proliferation and apoptosis by impinging on microtubule formation and stability (Fig. 7).

In summary, we identified Bcl-xl, Mcl-1 and Cyclin B1 as mediators of the antineoplastic effects of tivantinib. Our study provides a possible explanation for the preferential effect of tivantinib in c-MET-high patients but indicates that the beneficial clinical effect of tivantinib may not be predictable solely by c-MET, and prompts for the evaluation of the expression of other molecules, such as Mcl-1, Bcl-xl and Cyclin B1 as possible and potentially more reliable markers for patients' selection.

## MATERIALS AND METHODS

### Cell lines culture and reagents

Huh7, HepG2, Hep3B, Chang cells (hepatocellular carcinoma), TFK1 (human cholangiocarcinoma), DLD1 cells (human colon carcinoma), PL5 and PANC1 cells (human pancreatic carcinoma) were used for *in vitro* experiments. DLD1 colorectal cancer cells and two different syngenic *c-MET* exon 16 knock-out DLD-1 clones were provided by A. Bardelli and S. Arena and have been previously described [13]. All cell lines were cultivated in standard cell culture media as recommended by the providers. Tivantinib (ARQ197, provided by ArQule, Woburn MA, USA.) was dissolved in 100% DMSO and stored at −20°C. Tigatuzumab (CS-1008, by Daiichi Sankyo Pharma Development, Edison NJ, USA) was provided as a 10 mg/ml solution and stored at 4°C. Recombinant Human HGF was purchased from R&D (Minneapolis, MN USA).

### Cell viability assay

Cells were seeded onto 96-well plates at different cell densities to avoid overgrowth (0.6–3.5 × 10^3^/well) and were treated with increasing concentrations of tivantinib or vehicle. To investigate the effect of tivantinib on cell viability at low concentrations, cells were kept in culture for 6 days. At day 6 cells were washed with PBS, underwent osmotic-lysis in 100 μl ddH_2_O, and then incubated in 5% CO_2_ incubator for 45 minutes. Fluorescence was measured after addition of 0.2% Sybr green (Cytoflour Series 4000, Applied Biosystems, Framingham, MA USA).

### Colony formation assay

4–5 × 10^3^ cells were plated onto 6-well plates. After 24-h incubation with tivantinib cells were allowed to grow for 3 weeks. Colony formation was evaluated after the cells were fixed in 9% paraformaldehyde and stained with crystal violet for 30 minutes (Sigma, St. Louis, MO USA). Total cell colonies in each well were counted after being photographed.

### Apoptosis and cell cycle assay

8.0 × 10^4^−1.5 × 10^5^ cells were seeded in 12-well plates and treated after overnight incubation. Fluorescence activated cell sorting (FACS– Accuri-C6 flow cytometer, BD Biosciences San Jose, CA USA) was performed to detect the sub-G1 cell fraction to determine apoptosis and the respective different phases of cell cycle after propidium iodide (PI) staining as previously described [27]. In addition, apoptosis was assessed morphologically by Hoechst 33342 staining and fluorescence microscopy.

### Western blot and caspase activity assays

Cells growing in Petri dishes were collected and lysed in cell lysis buffer (Cell Signaling Technology, Boston MA USA) containing 20 mmol/L Tris-HCL (PH7.5), 150 mmol/L NaCl, 1 mmol/L Na_2_EDTA, 1 mmol/L EGDA, 1% Triton, 2.5 mmol/L sodium pyrophosphate, 1 mmol/L β-glycerophosphate, 1 mmol/L Na_3_VO4, 1 μg/ml leupeptin, and 1 mmol/l phenylmethane sulphonyl fluoride. Membranes were incubated overnight at 4°C with specific antibodies in TBS-T (TBS-0.1% Tween20) and subsequently for one hour with the appropriate horseradish-peroxidase-conjugated anti-rabbit/mouse secondary antibody. The following mouse monoclonal antibodies were used: Anti-CyclinB1 (Santa Cruz Biotechnology, Dallas Texas USA), anti-Caspase-8, anti-β-actin (both from Cell Signaling Technology, Boston MA USA). The following rabbit polyclonal antibodies were used: anti-Caspase-3, anti-PARP, anti-Bid, anti-Bad, anti-Bcl-2, anti-Bcl-xl, anti-Bax, anti-Mcl-1 (all from Cell Signaling Technology), anti-c-MET, anti-phosphorylated c-MET (Tyr1349 and Tyr1234/1235). For Caspase-3/7 activity assays *the* Apo-ONE® Reagent kit (Promega, Madison WI USA) was used according to the instructions of the manufacturer. Actin was used for control of appropriate protein load for each membrane. In figures, one actin loading control has been exemplarily shown.

### Small interfering RNA and transfection

Cyclin B1, Bcl-xl, Mcl-1 and c-MET were silenced by small interfering RNA (siRNA) or non-coding siRNA. Specific siRNA SMARTpool® sequences consist of 4 pooled 21-nucleotide RNA oligonucleotides forming a 19-bp duplex core with 2-nucleotide 3 overhang and were purchased from Dharmacon, Inc. (Thermo Scientific, Rockford IL USA). For transfection the DharmaFECT4 reagent (Thermo Scientific, Rockford IL USA) was used according to the manufacturer's instructions.

### Statistical analysis

Statistical analysis was performed by using IBM SPSS Statistics (SPSS Inc, IL USA). Comparison of means was performed by two-tailed Student's test. Results are expressed as mean and standard deviation (SD) of at least 3 independent experiments. *P* values < 0.05 were regarded as statistically significant.

## SUPPLEMENTARY FIGURES


