# Supplementary material for: Tivantinib (ARQ 197) affects the apoptotic and proliferative machinery downstream of c-MET: role of Mcl-1, Bcl-xl and Cyclin B1
Source: Oncotarget. 2015 Jun 10;6(26):22167–78. doi: 10.18632/oncotarget.4240 (PMC4673154; doi:10.18632/oncotarget.4240)
Supplement: Supplementary file 1 [file oncotarget-06-22167-s001.pdf]

SUPPLEMENTARY FIGURES

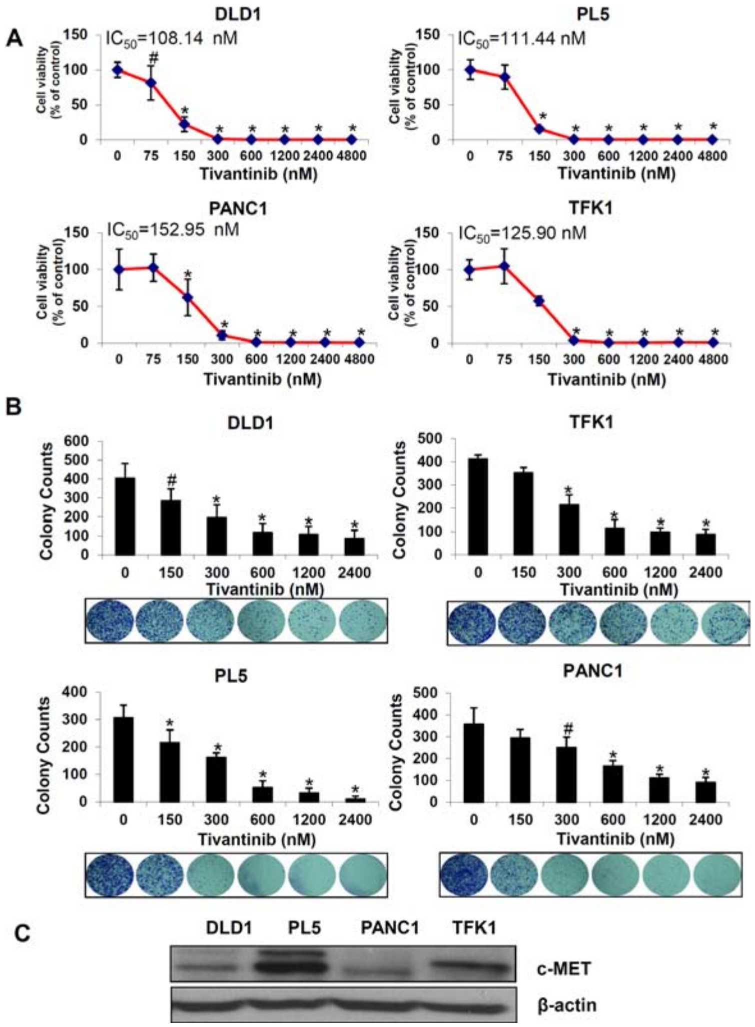

Supplementary Figure S1: Effect of tivantinib on proliferation and colony formation of colon cancer, cholangiocarcinoma and pancreatic cancer cells. A. 0.5–3.5 × 10^3 cells (DLD1, TFK1, PL5 and PANC1) per well were seeded onto 96-well plates. Viability assay was performed as described in figure 1. **B.** Colony formation assay of the same cell lines as described in the legend to figure 1. *$p < 0.01$; #$p < 0.05$ in comparison to control treated cells. **C.** Western blot analysis of c-Met in the cell lines used in the previous panel.

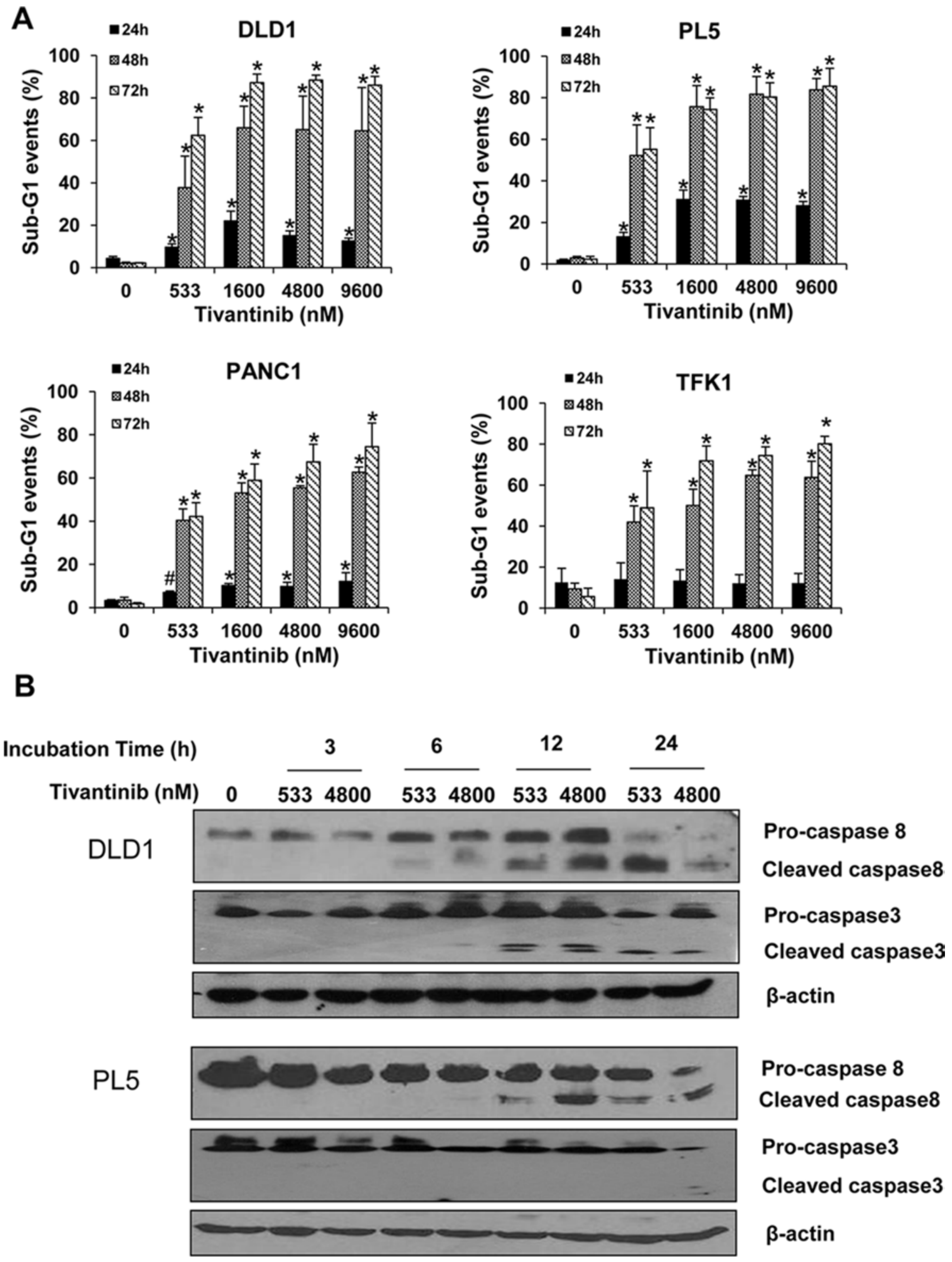

Supplementary Figure S2: FACS-analysis based quantitation of apoptosis after PI-staining and caspase 3 analysis of tivantinib-treated cells. A. Apoptosis was measured by the quantification of sub-G1 events after propidium iodide staining in pancreatic cancer cells (PANC1, PL5), colorectal cancer cells (DLD1) and cholangiocellular carcinoma cells (TFK1). Assays were replicative of at least three independent experiments. *$p < 0.01$; #$p < 0.05$ in comparison to control treated cells. **B.** Assessment of caspase 8 and caspase 3 cleavage by western blot in DLD1 and PL5 cells.

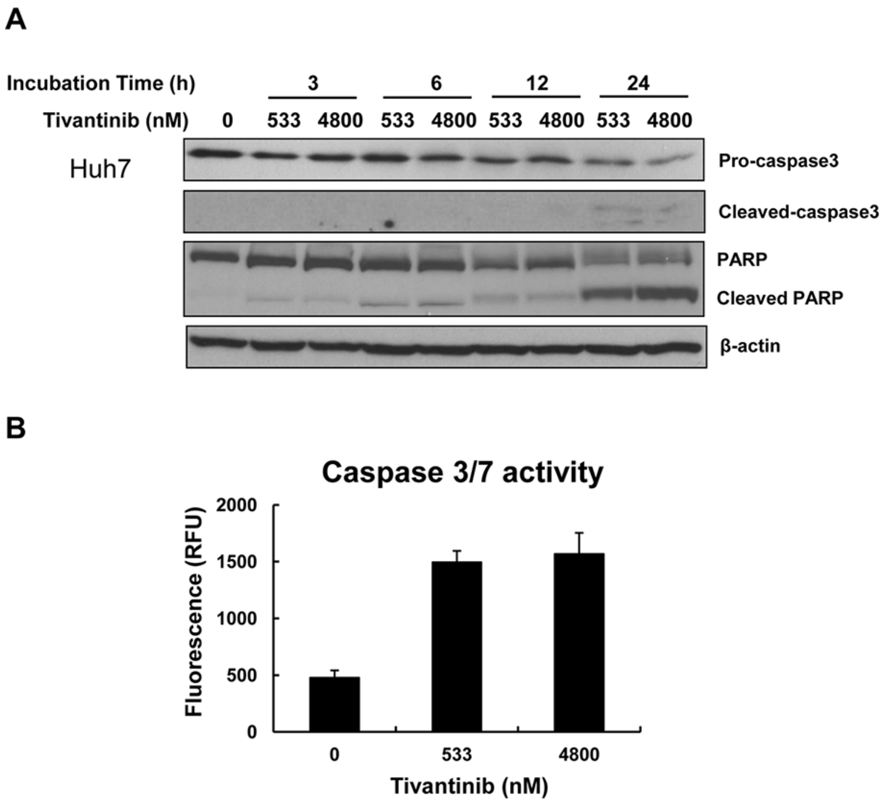

Supplementary Figure S3: Tivantinib causes caspase–dependent apoptosis. A and **B.** Effect of tivantinib on caspase activation and PARP as judged by western blot (A) or functional assessment of caspase 3/7 activation in Huh7 cells (B).

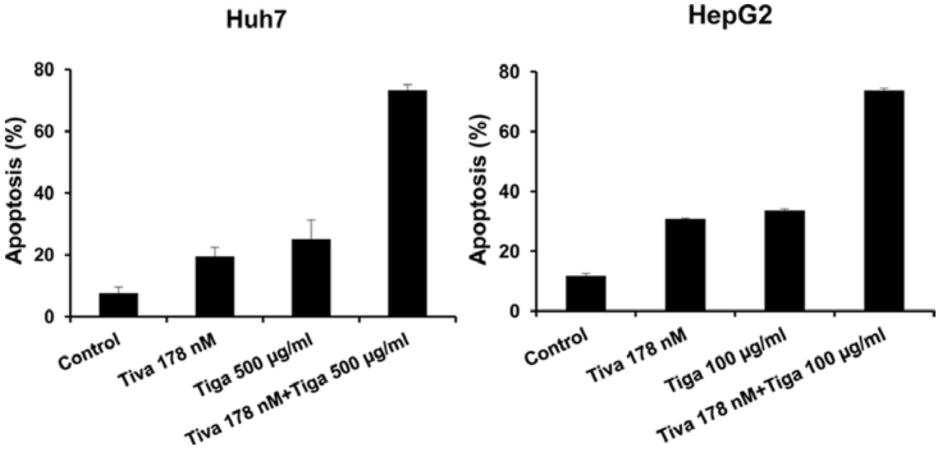

Supplementary Figure S4: Tivantinib treatment sensitizes HCC cells to the apoptotic effect of tigatuzumab. FACS analysis was performed after 24 hours to measure the fraction of apoptotic cells after propidium iodide staining. Graphs show average rates of apoptosis and standard deviation of one representative experiment, performed in triplicate and repeated at least three times. Combined application of tivantinib and tigatuzumab caused a 2.9 ± 0.7 fold increase of apoptosis in Huh7 cells and a 1.5 ± 0.1 fold increase of HepG2 cells vs. the administration of these agents alone.
